# Ternary Resistance Switching Memory Behavior Based on Graphene Oxide Embedded in a Polystyrene Polymer Layer

**DOI:** 10.1038/s41598-017-04299-z

**Published:** 2017-06-21

**Authors:** Yanmei Sun, Dianzhong Wen, Xuduo Bai, Junguo Lu, Chunpeng Ai

**Affiliations:** 1 HLJ Province Key Laboratories of Senior-education for Electronic Engineering, Heilongjiang University, Harbin, 150080 China; 2 Communication and Electronics Engineering Institute, Qiqihar University, Qiqihar, 161006 China; 3 School of Chemistry and Materials Science, Heilongjiang University, Harbin, 150080 China

## Abstract

Nonvolatile ternary memory devices were fabricated using the composite of polystyrene (PS) and graphene oxide(GO) as active layers, which have an reliable intermediate state. The current-voltage (*I*-*V*) curves of the indium tin oxide (ITO)/PS+GO/Al device under the external applied voltages exhibited current tri-stability with three conductivity states, which clearly revealed ternary memory performance. Under the stimulus of the external voltage, a stable intermediate conductivity state was observed. In the write-read-erase-read test, the ITO/PS+GO/Al device exhibited rewritable, nonvolatile, ternary memory properties. The resistance as functions of the time indicated that three conductivity states held for 2 × 10^5^ s, suggesting that the good stability of the ITO/PS+GO/Al devices. HRTEM and XPS observation indicated that the Al top electrode reacted with oxygen within in GO.

## Introduction

The rapid growth of intelligent portable electronic products put forward a great demand for developing next generation nonvolatile memory devices with high density storage, high endurance, simple cell structure, fast operating speed and lower consumption^1, 2^. It becomes obvious that traditional silicon-based storage systems are at the limit of their downscaling and memory capacities^3, 4^. For the past few years, organic memory devices are widely discussed and investigated due to their advantages of low cost, light weight, solution processability, and availability of structural varieties^5–9^. At present, most organic resistive switching memory devices show two conductivity states (that are ON and OFF states) in response to the external applied voltage^10–14^. The data storage capacity in such devices are only 2^*n*^. Therefore, to achieve ultrahigh density data storage, organic materials with multilevel stable conductivity states, which can realize the increasing data storage capacity of 3^*n*^ or larger, are highly desirable^15–17^.

Graphene, comprising one monolayer of carbon atoms packed into a two-dimensional honeycomb lattice^18, 19^, has outstanding properties, for instance, superior mobility^20^, heat stability^21^, current-carrying capabilities^22^, and room temperature ballistic transport^18^, which make it promising in electric, optoelectronic, and photonic device materials. The graphene sheets, holding the post of charging and discharging media, have been remarkable on account of their being great potential candidates in hybrid nanocomposites-based memory devices. The hybrid composites were widely used in memory device on account of their excellent mechanical properties and low cost. At first, polymers were just utilized as matrix materials for small molecules^23–25^. Supplementary components like organic molecules or metal particles in polymer matrix were used as electron donors or acceptors to result in charge transfer complex formation^13, 26^. Later, the electro-active molecules like carbon nanotubes^6^ and graphene oxide (GO)^27–29^ were mixed with the “donor-containing polymer” matrix to research the resulting memory characteristics. Notwithstanding, some materials have been indicated to exhibit “0”, “1”, and “2” three states and have high data storage capacity of more than 2^*n*^ ^30–37^. Recently, Wu *et al*. reported a tristable resistive memory based on single layer graphene/insulating polymer multi-stacking layer^35^. A highly reproducible multilevel resistive memory device utilizing GO sheets/polyimide hybrid composite has been demonstrated by the same group^37^. The stable triple state memory characteristics in quadruple layers of Al/BCNT-nanocomposites/NCNT- nanocomposites/Al was reported by Hwang group^38^. Kim *et al*. by dip-coating method using ITO/RGO/ITO structures realized multi-level resistive switching behavior in the 00, 01, 10, and 11 states by varying the pulse height from 2 V to 7 V^39^. This result was remarkable in the organic multilevel resistive switching device field and evoked our attention for kinds of material in fabricating multilevel resistive switching memory device. However, there are still some puzzling problems remaining, such as, what is the critical factor that led to the multilevel resistive switching memory and what kinds of polymer composites own this property? In this article, we illustrate nonvolatile ternary memory devices fabricated from the facile solution casting of GO-composites blended into polystyrene (PS) matrix.

## Experimental section

The memory device was fabricated on an indium-tin-oxide (ITO) glass (6–9 Ω·sq^−1^) substrate. The ITO was pre-cleaned sequentially with deionized water, acetone, and methanol by ultrasonication for 20 min respectively. 2 ml chlorobenzene solution of PS (10 mg/ml) blending with 0.6 ml GO (10 mg/ml) in ethanol, which was sonicated for 40 min to form homogeneous dispersions. Afterwards, the GO + PS composite solution was spin-coated onto the ITO substrate, followed by drying at 60 °C in vacuum oven for 8 h. Finally, Al electrodes about 300 nm thick were thermally evaporated on the composite layers at a pressure of 10^−7^ Torr through a 300 μm diameter shadow mask. To explore the electrical behavior of the ITO/PS+GO/Al device, a external voltage was applied to the Al electrode. The compliance current was set as 0.1 A to avoid the ITO/PS+GO/Al device hard breakdown, and the ITO electrode was grounded.

## Results and Discussion

**Figure 1:**
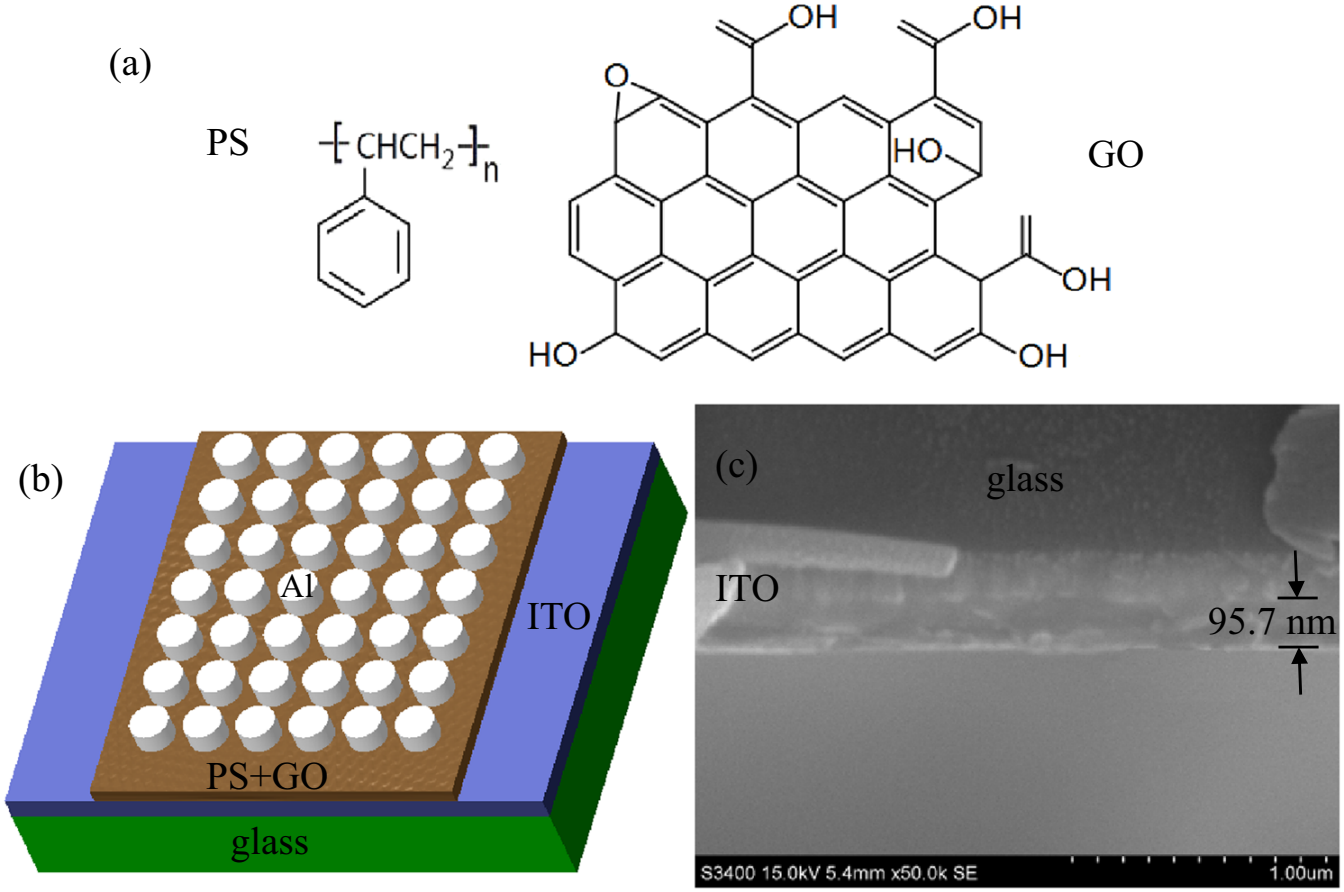
(**a**) Molecular structures of PS and GO. (**b**) The schematic diagram of the structure for the ITO/PS+GO/Al devices. (**c**) The SEM image of GO embedded in a PS layer.

Figure 1(a) shows the molecular structures of PS and GO. A schematic diagram of the structure for the ITO/PS+GO/Al devices used in this study is shown in Fig. 1(b). Figure 1(c) shows an scanning electron microscopy (SEM) image of the GO blended with a PS matrix.

**Figure 2:**
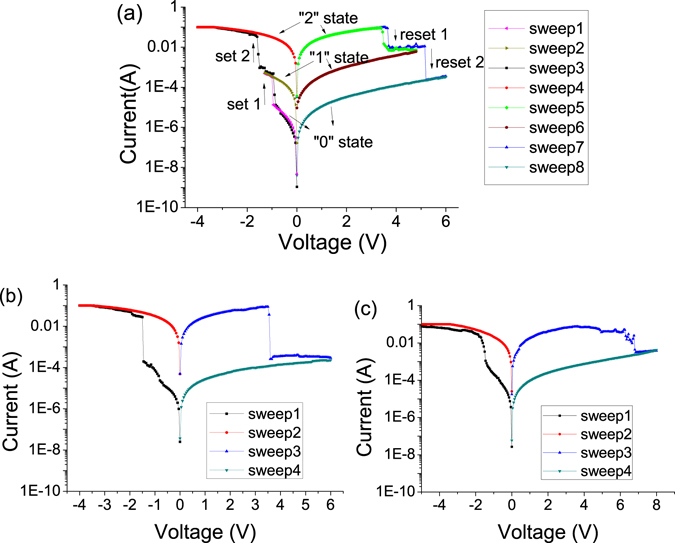
(**a**) The *I*-*V* characteristics of the ITO/PS+GO/Al device. (**b**) The *I*-*V* characteristics of the ITO/GO/Al device. (**c**) The *I*-*V* characteristics of the ITO/PS/Al device.

The current-voltage (*I*-*V*) curves of the ITO/PS+GO/Al device were gathered by Keithley 4200 semiconductor parameter analyzer. Figure 2(a) exhibits the *I*-*V* characteristics of the ITO/PS+GO/Al device fabricated from the composite thin films of PS and GO. In the first sweep from 0 to −1.3 V, a sudden current increases happened at −0.85 V of threshold switching voltages, the results showed that the transitions from a low-conductivity (“0”) state to an intermediate-conductivity (“1”) state. The cell held this “1” state in the subsequent the second voltage scan from 0 to −1.3 V. Sweep 3 was measured in another cell of the ITO/PS+GO/Al device over a voltage scan range of 0 to −4.0 V and exhibited two sudden current increases at −0.8 and −1.55 V of threshold switching voltages, demonstrating the transitions from a “0” state to an “1” state and then to a high-conductivity (“2”) state. The “2” state was held during subsequent the fourth sweep from 0 to −4 V. These two sudden current increases stand for the process of “writing” in memory device. When the applied voltage was swept from 0 to 4.8 V in the fifth sweep, at the switching threshold voltages of 3.5 V, the sudden current decrease took place, manifesting a transition from the “2” state to “1” state The cell held in this “1” state during subsequent the sixth sweep from 0 to −4.8 V. Sweep 7 was measured in another cell of the ITO/PS+GO/Al device in a voltage range of 0 to −6 V and exhibited two sudden current decreases at 3.7 and 5.2 V, demonstrating the transitions from “2” state to “1” state and then to “0” state. This two sudden current decreases can serve as the “erasing” process for ITO/PS+GO/Al device. The “0” state was maintained during subsequent the eighth sweep from 0 to 6 V. The *I*-*V* characteristics of the ITO/PS+GO/Al devices exhibited that there were two intermediate states with nonvolatile ternary memory behaviors. This result indicates that the ITO/PS+GO/Al device could be applied in three-value logic systems.

In order to clarify the effect of GO and PS on the resistive switching, ITO/GO/Al and ITO/PS/Al were prepared and the resistive switching of pure GO and pure PS was investigated. Figure 2(b,c) shows the *I*-*V* curve of ITO/GO/Al and ITO/PS/Al device, which exhibited the similar resistive switching behaviors to the ITO/PS+GO/Al device. Nonetheless, for ITO/GO/Al device, the low-conductivity state was abruptly converted to high-conductivity state at −1.70 V directly, and the high-conductivity state was suddenly switched to low-conductivity state at 3.65 V directly without the appearance of an intermediate-conductivity state as shown in Fig. 2(b). This clearly indicated that the ternary resistance switching memory behavior of the ITO/PS+GO/Al device was attributed to the presence of PS polymer. Figure 2(c) shows the *I*-*V* curve of ITO/PS/Al device, which exhibit small ON/OFF ratio. In contrast to ITO/GO/Al device, both the set and reset processes were gradual. Upon sweeping an external voltage, the current changed gradually from low-conductivity state to high-conductivity state, and the high-conductivity state was retained until an opposite voltage was applied. In our case, it is reasonable to postulate that the presence of PS modulate the electrical behavior of GO and change the charge transport properties. The chemical structure of polymer thin film can determine the resistive switching properties of the device^40^. So this difference in behaviors may be the result of a change in the chemical structure of the active layer upon mixing two chemical composition. PS is made out of saturated carbon chain with branched chains of carbon hydrogen bonds; while the mixed polymer layer contains PS chains in contact with GO, which are rich in hydroxyl groups. GO can provide the carbon-based network. Furthermore, oxygen species and electron traps are easily generated in GO, and they can construct electron hopping path which changes the resistance state^41^. The PS can act as charge blocking material. The hydrogen atoms in the PS at the composite system may be transferred to various oxygen-related groups in GO and residual water, which may affect the value of current and cause a change of resistance states.

**Figure 3:**
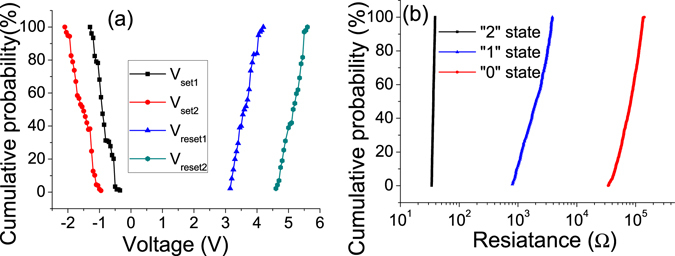
(**a**) The statistical distributions of the *V*_set1_, *V*_set2_, *V*_reset1_ and *V*_reset2_. (**b**) The statistical distributions of the *R*_0_, *R*_1_ and *R*_2_.

In order to verify the switching stability of the ITO/PS+GO/Al devices, cumulative probability distributions for *V*_set_, *V*_reset_ *R*_0_, *R*_1_ and *R*_2_, are plotted. When the ITO/PS+GO /Al device was carried out cyclic programming operations, *V*_reset1_ and *V*_reset2_ distribute in a range of 3.15~4.2 V and 4.6~5.6 V, respectively, while *V*_set1_ and *V*_set2_ show a distribution in a range of −0.35~ −1.3 V and −0.95~ −2.1 V as shown in Fig. 3(a), respectively. All of the operation parameters for the ITO/PS+GO/Al devices featured a standard deviation (*Δ*) to mean (*μ*) ratio, and *V*_set1_, *V*_set2_, *V*_reset1_ and *V*_reset2_ with *Δ*/*μ* values of −0.35641, −0.23112, 0.08926, and 0.06203, respectively. The overlapping of switching threshold voltages between the *V*_set1_ and *V*_set2_ and the small separation between the *V*_reset1_ and *V*_reset2_ are very adverse to the uniformity of write/read/ erase operation, it must be improved in the future to enable practical application in electronics. The statistical analysis generates the average values of *V*_set1_,*V*_set2_, *V*_reset1_ and *V*_reset2_ as −0.83, −1.53, 3.67 and 5.1 V, respectively. Figure 3(b) exhibits the resistance distributions of the *R*_0_, *R*_1_ and *R*_2_. The three different conductive states can be clearly distinguished. The *Δ*/*μ* values of *R*_0_, *R*_1_ and *R*_2_ were 0.03947, 0.3499 and 0.4361 respectively.

**Figure 4:**
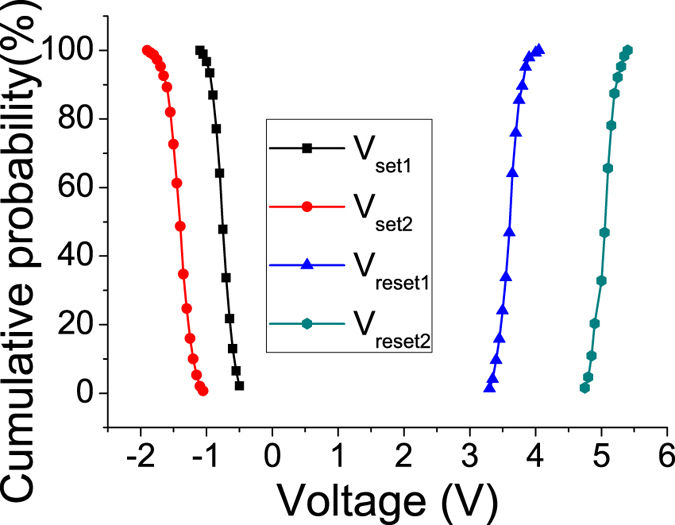
The distributions of threshold switching voltage with an diameter of 60 μm of top Al electrode.

In order to study the influence of electrode size on resistive switching behavior of ITO/PS+GO/Al device, the diameter of top Al electrode was downscaled to 60 μm. When the diameter of top Al electrode reduced from 300 μm to 60 μm, the key parameters, including the current, the resistance ratio and average switching voltage do not change obviously from cell to cell. In contrast, the distributions of threshold switching voltage with an diameter of 60 μm are found to be fluctuated in a very narrow range compared to that of the device with the top Al electrode diameter of 300 μm as shown in Fig. 4. The *Δ*/*μ* values of ITO/PS+GO/Al devices with the diameter of 60 μm of top Al electrode for *V*_set1_, *V*_set2_, *V*_reset1_ and *V*_reset2_ were −0.24341, −0.18097, 0.06405 and 0.042184, respectively. This results show that the reduced size of top Al electrode obviously helps to improve the uniformity of the resistive switching properties and is significant to the fabrication of high-density memory arrays in practical application.

**Figure 5:**
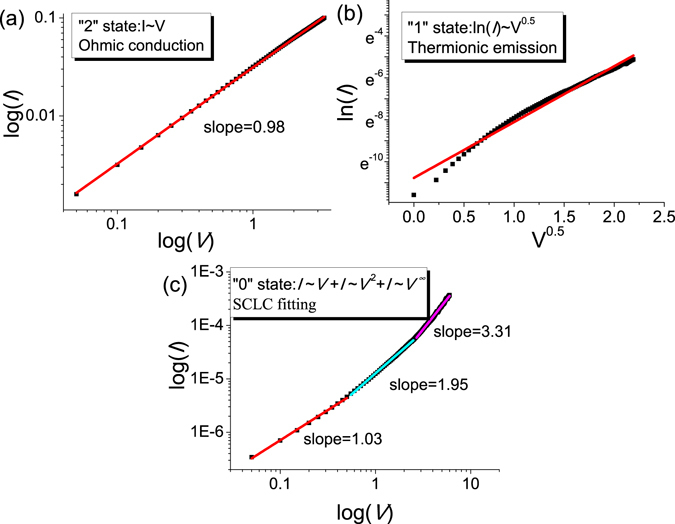
Analysis of *I*-*V* characteristics for the ITO/PS+GO/Al device: (**a**) Ohmic conduction behavior in the “2” state; (**b**) thermionic emission in the “1” state; (**c**) SCLC model in the “0” state.

In order to make it clear that the resistive switching mechanism of the nonvolatile ternary memory behavior of ITO/PS+GO/Al device, *I*-*V* curves were fitted with appropriate charge transport models. The *I*-*V* relationship in the “2” state showed Ohmic conduction behavior with a slope of 0.98 as shown in Fig. 5(a), demonstrating the formation of filament paths during the set process. For the *I*-*V* curve in the “1” state as shown in Fig. 5(b), the plot of ln(*I*)~*V* ^0.5^ was well fitted to a line. Such a linear relation indicates that the conduction mechanism in the “1” state could be regarded as the thermionic emission process^42, 43^. This exhibited that the conducting carriers were the free carriers, which were thermally generated and transfered via Schottky emission. The conduction is dominated by charge injection^11^. By comparison, the fitting result at “0” state as shown in Fig. 5(c) showed that the charge transport behaviors follow the trap-controlled space charge limited conduction (SCLC) model^44–47^. The SCLC model contains three different conductive regions as follows: (i) a low-voltage region where the *I*-*V* curve shows linear behavior with a slope of 1.03 (*I*∝*V*), which conform to the Ohmic conduction mechanism, (ii) a transition region where the current increases nonlinearly with voltage square dependence (*I*∝*V* ^2^) and the slope increases to 1.95, which is on the verge of the Child’s law, and (iii) a region labelled with a dramatic current increase where the slope is 3.31. The charge carriers injected from the electrodes to the organic composite layer increased with increasing applied voltage, leading to formation of space charges in the organic composite layer near interface. Therefore, space charge limited conduction was dominant. This behavior is consistent with previous reports on the interfacial effects^48, 49^, where the migration of oxygen vacancies in the vicinity of the interface drives resistive switching in various hetero-junctions. Under a negative electric field, oxygen vacancies with positive charges migrate away from the interface between active layer and Al, which widens the depletion layer, resulting in the low conductivity state. On the other hand, under positive voltage, the oxygen vacancies start moving toward the interface, which sets the device to high conductivity state. Therefore, the ITO/PS+GO/Al device exhibited different transport behaviors in different conductivity states: the Ohmic conduction mechanism at the “2” state, the thermionic emission mechanism in the “1” state, and the trap-controlled SCLC mechanism in the “0” state. The conductivity switching was brought about the charge transfer existing in the composite thin film. The different transport behaviors could be attributed to disparate charge transfer process occurring at different states.

**Figure 6:**
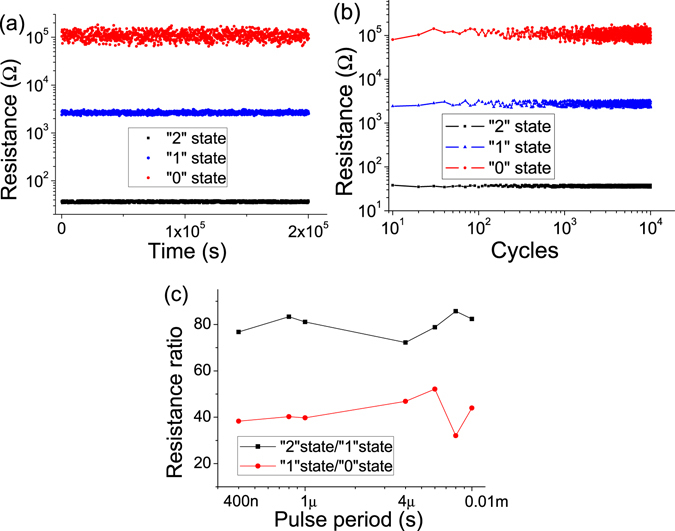
(**a**) Retention test of the ITO/PS+GO/Al device at reading voltage of 1 V. (**b**) Endurance performance of the ITO/PS+GO/Al device in the pulse switching mode. (**c**) Dependence of resistance ratio on pulse period in ITO/PS+GO/Al device.

The data retention and endurance performance are critical performances for memory devices. In order to measure the data retention ability of ITO/PS+GO/Al device, the “0”, “1” and “2” state were took notes at a reading voltage of 1 V. As shown in Fig. 6(a), the programmed “0”, “1” and “2” state were remained at the same order of magnitude without significant degradation for a duration of 2 × 10^5^ s. For exploring the endurance performance, the ITO/PS+GO/Al device was switched to “2” state by a negative pulse of −4 V/8 μs (the pulse duration and period are 4 and 4 μs, respectively) and was switched to “1” state by a positive pulse of 4.5 V/8 μs, and then was switched to “0” state by a positive pulse of 6 V/8 μs, after each pulse, the resistance was read out at 1 V. As shown in Fig. 6(b), the stored states was quite stable when the cycle number up to 10^4^ times. The programming speed was another key criterion to evaluate a memory device, in previous works, the fast switching speed of < 5 ns has been reported^50^. In order to evaluate the switching speed of ITO/PS+GO/Al device, the dependence of the resistance ratio on pulse period was researched as shown in Fig. 6(c). During pulse period reduced to 400 ns from 0.01 ms, the resistance ratio of “2” state/“1” state and “1” state/“0” state were basically stable. This result indicates the ITO/PS+GO/Al device operates well with very fast operation speed.

**Figure 7:**
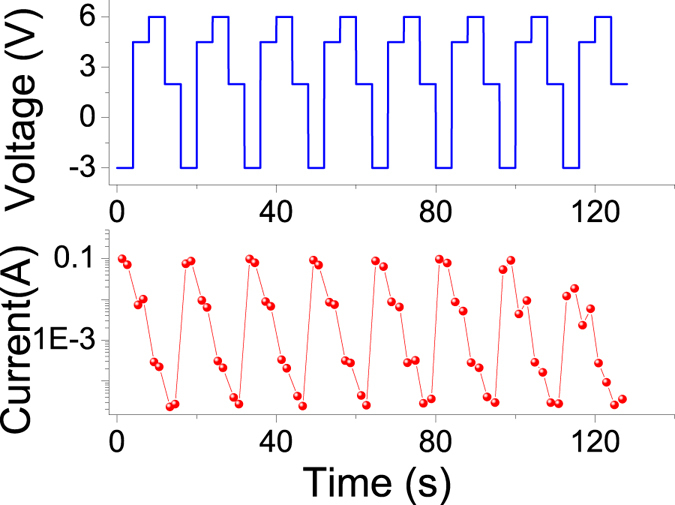
WRER test for the ITO/PS+GO/Al device.

The “write-read-erase-read” (WRER) test was explored to evaluate the resistive switching performances of the ITO/PS+GO/Al devices and to assess the capability of the ternary resistive switching memory devices serve for rewritable memories. The set pulse of −3 V was used to turn the ITO/PS+GO/Al device to the “2” state (Fig. 7). The reset 1 pulse of 4.5 V was used to turn the ITO/PS+GO/Al device back to the “1” state, and the reset pulse of 6 V was used to turn the ITO/PS+GO/Al device back to the “0” state. Afterwards, the reading pulses of 2 V were used to read data, a high current in around of 0.1 A at −3 V, an intermediate current about 0.01 A at 4.5 V, and a low current state in around of 2 × 10^−5^ A at 2 V, to distinguish the different conductivity states of the ITO/PS+GO/Al devices. The differences in the three conductivity states were clearly observed for up to 120 s.

**Figure 8:**
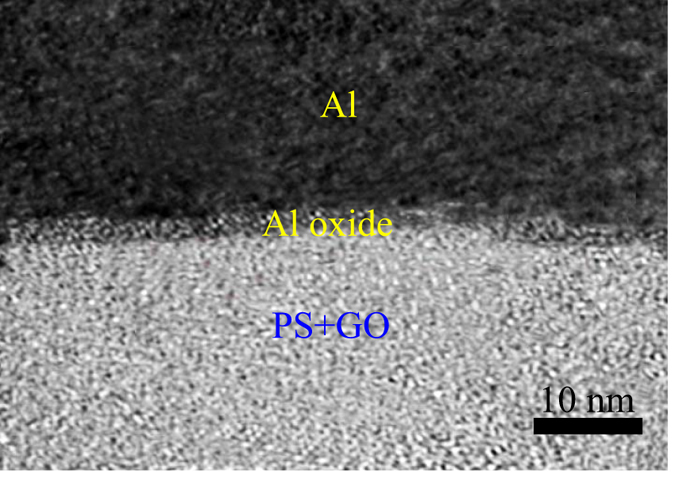
The cross-sectional HRTEM images of ITO/PS+GO/Al structures.

In many preceding works, such a resistive switching has been attributed to the interfacial conduction mechanism related to the formation of a thin aluminum oxide layer at Al top electrode^47, 49–52^. For this reason, Ag was used as the top electrode to replace Al on account of the work functions of Ag is similar to that of Al, but Ag has greater inertia from oxidation than Al. Our results suggest that when the top electrode was replaced by Ag, the device does not show resistive switching behavior. Therefore, the resistive switching in ITO/PS+GO/Al device should be connected with the Al top electrode. To research the conduction and resistive switching mechanisms in ITO/PS+GO/Al device, HRTEM experiments were performed, the cross-sectional TEM images of ITO/PS+GO/Al structures were showed in Fig. 8. This images indicated the formation of distinct interface layer between Al electrode and organic composite active layer, and the native oxide layer was clearly observed at the interface between Al electrode and PS+GO film.

**Figure 9:**
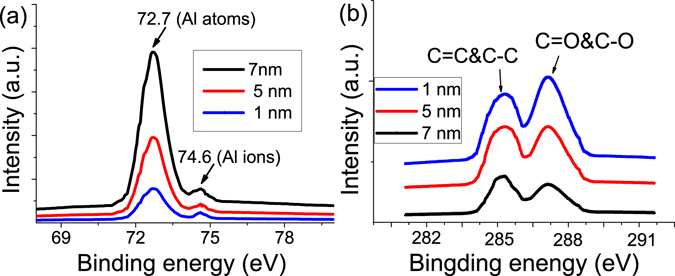
(**a**) The XPS spectrum of Al 2*p*; (**b**) The XPS spectrum of C 1*s*.

In addition, *in-situ* XPS experiments were also to verified above observations. *In-situ* XPS spectra were obtained when the top electrode Al were gradually deposited to 1 nm, 5 nm and 7 nm thickness on the PS+GO film. As shown in Fig. 9(a), except for the Al peak located at 72.7 eV, peaks at 74.6 eV (Al^3+^) were also observed in Al 2*p* spectra, which is clearly consistent with the HRTEM images. There are two potential sources of Al ions: oxidized Al formed by reaction with residual oxygen in the chamber, and reacted with GO by charge transfer between the Al and GO. In C 1*s* spectra, the peaks corresponding to C-O and C = O chemical bonds decreased as the thickness of Al layer increased as shown in Fig. 9(b). These results shows that the GO in surface of the composite films was reduced while the Al at the interface was oxidized. This result is consistent with the previously reported literature^41, 47, 53^.

**Figure 10:**
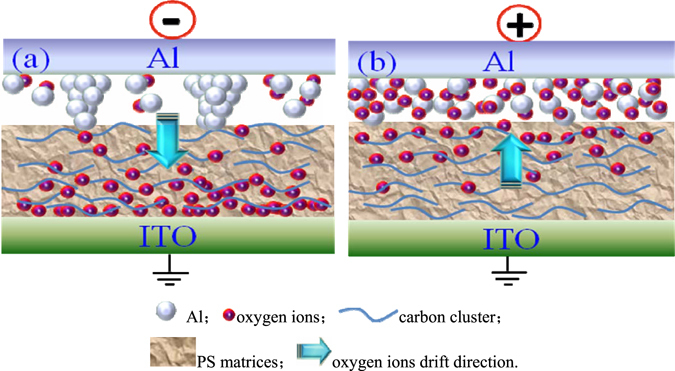
Schematic diagram of the resistive switching model for ITO/PS+GO/Al device. (**a**) The negative bias was applied; (**b**) The positive bias was applied.

The resistive switching mechanism of the ITO/PS+GO/Al device was schematically illustrated in Fig. 10. Spin-casting of the PS+GO composites on ITO substrates results in a conductive network with three dimensional and random orientation of GO in the PS matrix. An interface layer is formed through the chemical reaction between metal Al and oxygen ions within GO in composite layer. The large resistance due to this interface layer remain the ITO/PS+GO/Al device in the low conductivity state. When the negative bias was applied to top Al electrode as shown in Fig. 10(a), oxygen ions with negative charges migrate away from the interface between active layer and Al, the migration of oxygen ions leads to an Al-rich region in the interface. The Al-rich region crystallized along the top Al metal grains.Hence, the metallic conducting filaments were formed in the interface, and the total resistance was reduced, switching the ITO/PS+GO/Al device to high conductivity state. When the positive bias was applied to top Al electrode as shown in Fig. 10(b), the oxygen ions start moving toward the interface, Al conducting filaments were oxidized, the total resistance was increased, which converts the ITO/PS+GO/Al device to high conductivity state.

**Figure 11:**
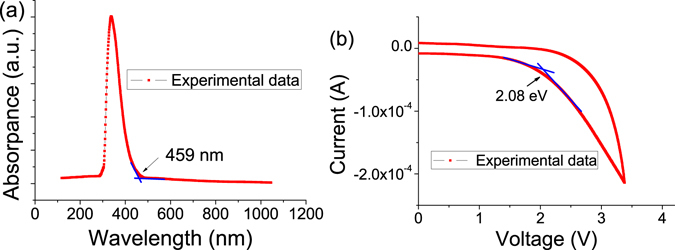
(**a**) UV-vis absorption spectra of GO film. (**b**) C-V sweeps of the GO film.

The bandgap of GO was known to be variable and affected by a number of factors^54, 55^. In order to determine the lowest unoccupied molecular orbital (LUMO) and highest occupied molecular orbital (HOMO) energy levels of GO in our device, the UV-vis absorption spectrum and cyclic voltammetry (*C*-*V*) sweeps for GO film were measured. Figure 11(a) shows the UV-vis absorption spectrum for GO film. A distinct absorbance band centered at around 337.5 nm was observed, with the absorption edge extending to a wavelength (ledge) of 459 nm, based on which the bandgap (*E*_g_) of the GO was calculated to be 2.70 eV. *C*-*V* sweeps of the GO film was exhibited in Fig. 11(b), based on this data, the GO shows an oxidation behavior with the oxidation peak at 2.08 eV. According to the above experimental data, the HOMO and LUMO energy levels of GO were evaluated, and the results are shown in Table 1.

**Table 1:** Optical and electrochemical properties of GO.

${{\boldsymbol{\lambda }}}_{max}{\boldsymbol{(}}{\boldsymbol{n}}{\boldsymbol{m}}{\boldsymbol{)}}$	${\lambda }_{{\rm{o}}{\rm{n}}{\rm{s}}{\rm{e}}{\rm{t}}}$(nm)	*E*_g_(eV)^*a*^	*E*_onset_(eV)	HOMO(eV) ^*b*^	LUMO(eV)^*c*^
337.5	459	2.70	2.08	−6.50	−3.80

^*a*^ *E*_g_ is estimated from the UV-vis absorption edge wavelength (ledge) using the Planck equation: band gap = $\frac{1240}{{\lambda }_{{\rm{o}}{\rm{n}}{\rm{s}}{\rm{e}}{\rm{t}}}}$ ^56^. ^*b*^The HOMO energy levels were determined from the *C*-*V* onset ionization potential (*E*_onset_) using ferrocene as the external reference (4.8 eV below the vacuum level): *E*_HOMO_ = −[*E*_onset_-*E*_ferrocene_ + 4.8)] (eV). *E*_ferrocene_ is determined to be 0.38 V *vs*. Ag/AgCl. ^*c*^ *E*_LOMO_ is determined from the equation: *E*_LOMO_ = *E*_HOMO_ + *E*_g_^57^_._

**Figure 12:**
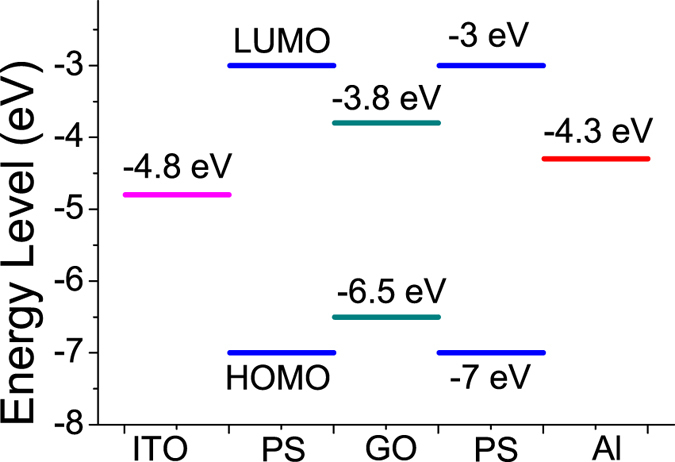
Energy band diagram of the ITO/PS+GO/Al device.

The energy band diagram of the ITO/PS+GO/Al device devices as shown in Fig. 12. The value of LUMO and HOMO of PS was acquired by literature^38, 58^. In addition, presence of various type defects in graphene is a well known fact^59^. Those two sudden current transition in set and reset progress for ITO/PS+GO/Al devices is probably attributed to various factors, such as: (i) the formation and rupture of conducting filaments at the top interface, not throughout the entire PS+GO composite film; (ii) The energy level difference between the PS matrix and the GO sheets forms potential wells which will produce an effect on charge transport and the varying filling degrees of potential wells in the active layer at different applied bias; (iii) the defect present in GO may have different potentials^59^.

In summary, nonvolatile ternary memory devices based on the composite thin films of PS and GO were illustrated. A stable intermediate state was observed under positive and negative applied voltages in the ITO/PS+GO/Al device. The fitted *I*-*V* curves indicated that the carrier transport mechanisms correlation with the memory behaviors of the ITO/PS+GO/Al devices were ascribed to SCLC, thermionic emission, and Ohmic conductions. The ITO/PS+GO/Al device exhibited a stable endurance of over 10^4^ cycles and long retention time of 2 × 10^5^ s. HRTEM and XPS analyses clearly demonstrated the reduction at the interface of the GO and oxidation of the top Al electrode. Nonvolatile ternary memory devices with a single organic composite layer bid fair to frontier applications in next generation memory devices with ultra-high density storage capacities.
